# The Influence of Annealing and Film Thickness on the Specific Properties of Co_40_Fe_40_Y_20_ Films

**DOI:** 10.3390/ma16062490

**Published:** 2023-03-21

**Authors:** Wen-Jen Liu, Yung-Huang Chang, Chia-Chin Chiang, Yuan-Tsung Chen, Yu-Chi Liu, Yu-Jie Huang, Po-Wei Chi

**Affiliations:** 1 Department of Materials Science and Engineering, I-Shou University, Kaohsiung City 840, Taiwan; jurgen@isu.edu.tw; 2 Bachelor Program in Interdisciplinary Studies, National Yunlin University of Science and Technology, 123 University Road, Section 3, Douliou, Yunlin 64002, Taiwan; changyhu@yuntech.edu.tw; 3 Department of Mechanical Engineering, National Kaohsiung University of Science and Technology, Kaohsiung City 80778, Taiwan; ccchiang@nkust.edu.tw; 4 Graduate School of Materials Science, National Yunlin University of Science and Technology, 123 University Road, Section 3, Douliou, Yunlin 64002, Taiwan; dream030709@gmail.com (Y.-C.L.); v796189000@gmail.com (Y.-J.H.); 5 Institute of Physics, Academia Sinica, Nankang, Taipei 11529, Taiwan; jacky01234567891@hotmail.com

**Keywords:** X-ray diffraction (XRD), low-frequency alternating current magnetic susceptibility (χ_ac_), optimal resonance frequency (f_res_), surface energy, adhesion, electrical properties, transmittance

## Abstract

Cobalt Iron Yttrium (CoFeY) magnetic film was made using the sputtering technique in order to investigate the connection between the thickness and annealing procedures. The sample was amorphous as a result of an insufficient thermal driving force according to X-ray diffraction (XRD) examination. The maximum low-frequency alternate-current magnetic susceptibility (χ_ac_) values were raised in correlation with the increased thickness and annealing temperatures because the thickness effect and Y addition improved the spin exchange coupling. The best value for a 50 nm film at annealing 300 °C for χ_ac_ was 0.20. Because electron carriers are less constrained in their conduction at thick film thickness and higher annealing temperatures, the electric resistivity and sheet resistance are lower. At a thickness of 40 nm, the film’s maximum surface energy during annealing at 300 °C was 28.7 mJ/mm^2^. This study demonstrated the passage of photon signals through the film due to the thickness effect, which reduced transmittance. The best condition was found to be 50 nm with annealing at 300 °C in this investigation due to high χ_ac_, strong adhesion, and low resistivity, which can be used in magnetic fields.

## 1. Introduction

The high saturation magnetization (Ms) and low coercivity (Hc) are just two of the desirable soft magnetic properties of Cobalt Iron (CoFe) material as an over-alloy [1,2,3]. These characteristics of CoFe alloys are mostly used in composite permanent magnets, catalysts, and magnetic equipment. Additionally, soft magnetic materials can be used in thin films, which are then used in magnetic applications like sensors, actuators, magnetic reading heads, and magnetic random access memory (MRAM) [4,5,6,7,8]. However, CoFe films have fatal disadvantages at high temperatures, such as magnetic anisotropy degradation. After annealing at a particular temperature, thermal stability must be improved for the effective use of magnetic devices, so adding a third element may be one of the solutions to this problem [9,10,11]. When applying a magnetic film at a high temperature, it is vital to determine whether the magnetic film will be impacted by the thermal effect. The service life and durability of the magnetic components are important reference indices. Rare earth (RE) metals that are ferromagnetic typically have unique features that can be utilized to increase performance at high temperatures. The mechanical qualities of the alloy can be increased by including the rare earth element Yttrium (Y), which also has strong heat stability and corrosion resistance. The mechanical and toughness characteristics of the parent alloy may be enhanced by the Y addition. Additionally, this may facilitate the processing of the alloy and increase its resistance to oxidation under high temperatures [12,13,14,15,16]. Despite its abundance among rare earth elements, Y has received little attention. Significant characteristics are considerably improved by the addition of Y and heat treatment. By enhancing the exchange coupling effect, Y substitution can have an effect on remanence, magnetic energy, and thermal stability [17,18]. When pure Y is exposed to air, it spontaneously produces yttrium oxide (Y_2_O_3_), which has exceptional thermal stability, mechanical capabilities, as well as optical and electrical properties. The heat barrier layer is frequently made of Y_2_O_3_ film, which can also be utilized as a light anti-reflective layer [19,20,21,22]. In a magnetic tunneling junction (MTJ), a cobalt iron boron (Co_40_Fe_40_B_20_) thin film is typically encountered as a free or pinned layer. In order to enhance magnetic properties, Cobalt Iron Yttrium (CoFeY) films were mostly used in this work with the same Y/B ratio. The goal of this research is to see if annealed CoFeY thin films change in high-temperature environments by looking at their structure and magnetic characteristics. The creation of Co_40_Fe_40_Y_20_ films and whether adding Y can enhance the performance of these materials have been the main topics of our research up to this point. As a result, there has been minimal research in this field. It is critical to explore the properties of Co_40_Fe_40_Y_20_ films produced by sputtering at room temperature (RT) and annealing processes. At various thicknesses, the magnetic characteristics, adhesion, and optical performance of as-deposited and annealed films were also investigated. It is significant to note that when a CoFeB seed or buffer layer was replaced with a CoFeY film, the thermal stability of the CoFeY films was improved, making these materials more suitable for use in actual MTJ applications. Table 1 contains the abbreviations and full names of the proper nouns. In earlier studies, the magnetic and adhesion characteristics of CoFeY materials were compared to those of Cobalt Iron Vanadium (CoFeV), Cobalt Iron Tungsten (CoFeW), and Cobalt Iron Ytterbium (CoFeYb) in Table 2 [23,24,25,26,27]. In contrast to previous CoFeY films that were sputtered on Si(100) substrates to study magnetic and adhesion outcomes, the goal of this research is to produce thin films on glass substrates and also investigate optical transmittance properties. Additionally, contact angle measurements are a suitable and simple way to determine the surface energy using water and glycerol when taking into account the application of the film, which is a dominant factor in molecular interaction at the surface [28]. In most cases, researchers have emphasized the properties in light of the magnetic hysteresis curve, Ms, and Hc [29,30,31]. However, it will be interesting to learn how the variation of surface energy and optical transmittance can be correlated with the magnetic properties of the deposited films.

## 2. Materials and Methods

CoFeY films with thicknesses varying from 10 nm to 50 nm were sputtered at RT on a glass substrate by using a magnetron sputtering direct current (DC) approach. The investigations were carried out on the as-deposited films and after various annealed treatments at 100 °C, 200 °C, and 300 °C for 1 h in an Argon (Ar) environment. The Ar working pressure was 3 × 10^−3^ Torr, while the base pressure of the chamber was 3 × 10^−7^ Torr. There was 2.5 × 10^−3^ Torr of pressure in the ex situ annealed state. The experimental target is made of a variety of sintered materials. The distance between the substrate and the target is 30 cm. The target dimensions are 3 inches in diameter and 2 mm thick. The intended alloy composition for CoFeY was 40% Co, 40% Fe, and 20% Y. CoFeY targets were synthesized using pure metals and purchased from Gredmann Taiwan Ltd. A powder mixture was made using 99.9% pure elemental Co, Fe, and Y. The original factory has certified the composition ratio of the target material to test the composition. The power density per square centimeter was 1.65 W/cm^2^, and the deposition rate was 1.2 nm/min. The structure was determined using grazing incidence X-ray diffraction (GIXRD) patterns created with CuKα1 (PAN analytical X’pert PRO MRD) and a low angle diffraction incidence of approximately two degrees. Energy dispersive X-ray spectroscopy (EDX) was used for the determination of elemental composition. Atomic force microscopy (AFM) was used to analyze the surface morphology and calibrate the accurate thickness by the AFM height difference method. The in-plane magnetic susceptibility was examined using the low-frequency alternate-current magnetic susceptibility (χ_ac_) analyzer (XacQuan). The standard sample was first calibrated by the χ_ac_ analyzer with an external magnetic field. The operating frequency ranged from 10 to 25,000 Hz. It was found that magnetic susceptibility and frequency are related using an χ_ac_ analyzer. The χ_ac_ analyzer measures the optimal resonance frequency (f_res_), which is the frequency of maximum χ_ac_. The hysteresis loops and electrical properties of Co_40_Fe_40_Y_20_ films were studied by vibrating sample magnetometer (VSM) and four-point probe measurement. The contact angles (θ) of the CoFeY film were measured using deionized (DI) water and glycerol as testing liquids (CAM-110, Creating Nano Technologies, Tainan, Taiwan). The amount being tested is represented by the liquid drop that appears through the pinhole and falls to the film’s surface. The contact angle is used to compute the surface energy [32,33,34]. A spectrum analyzer was used to measure the transmittance using visible light with wavelengths ranging from 500 nm to 800 nm. The MTS Nano Indenter XP with a Berkovich tip and continuous stiffness measurement (CSM) techniques were measured the hardness and Young’s modulus. Before the indenter was gradually removed from the surface, the loading was reduced to 10% of the maximum load. Ten distinct measurements were made by the indenter for each sample.

## 3. Results

### 3.1. XRD, EDX, and AFM Morphology

**Figure 1:**
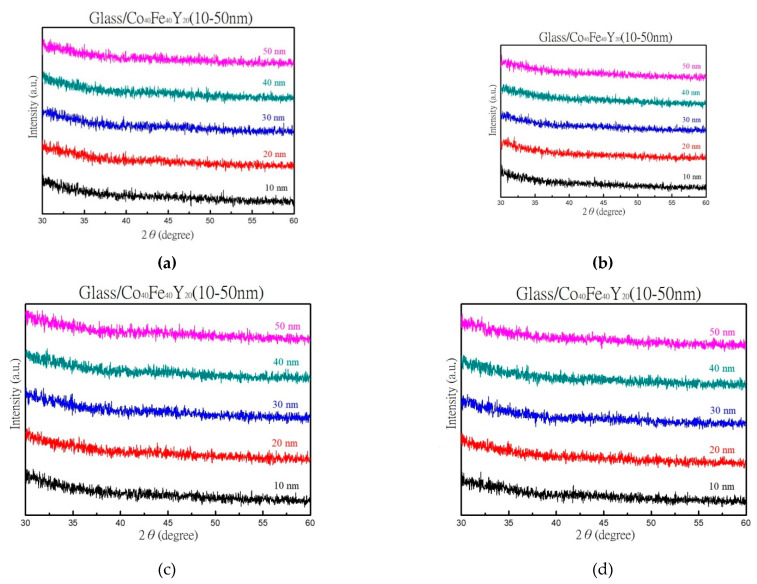
XRD patterns. (**a**) RT, (**b**) after annealing at 100 °C, (**c**) after annealing at 200 °C, (**d**) after annealing at 300 °C.

As-deposited and annealed XRD patterns of various thicknesses are depicted in Figure 1. Figure 1a displays a peak plot of the as-deposited condition, and Figure 1b–d display a plot of the film after it has been annealed at corresponding temperatures of 100 °C, 200 °C, and 300 °C. The above figures show that the film structure is amorphous. According to the literature, the growth of alloyed films also reveals an amorphous state [35]. Furthermore, it is possible to draw the finding that grain development is not sufficiently supported by a thermal driving force [36,37].

The composition of the CoFeY films, as discovered by EDX analysis at 50 nm, is displayed in Table 3. However, due to material loss during the sputtering technique’s transport from the target to the substrate, the stoichiometry of the film and target may not be the same.

**Figure 2:**
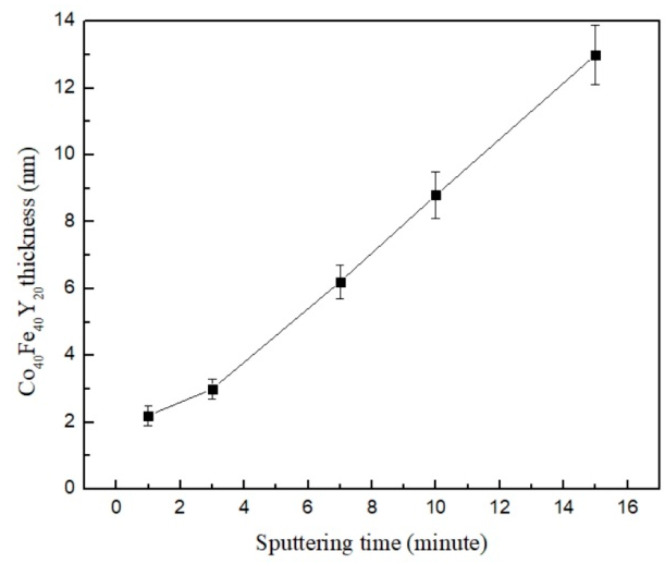
The corresponding thickness of associated sputtered time.

In order to calibrate the precise thicknesses, Figure 2 explores the corresponding thickness for the associated sputtering time. The linear relationship shown in this diagram suggests that thicker films are produced by longer sputtered times.

**Figure 3:**
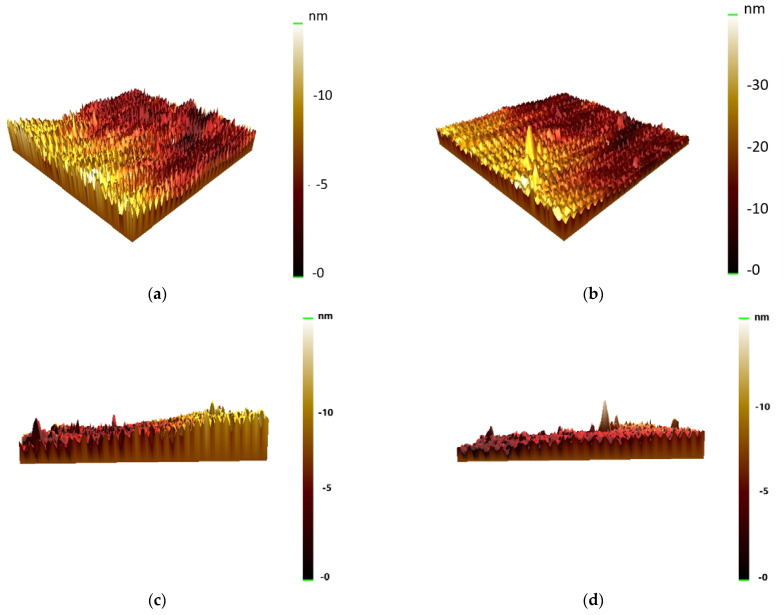
AFM surface morphology at RT at (**a**) 10 nm and (**b**) 40 nm. AFM profiles across the film edge at (**c**) 10 nm and (**d**) 40 nm.

AFM was used to observe the degree of film coverage, which is shown in Figure 3a,b. From the result, the incomplete coverage of the 10 nm film and the full coverage of the 40 nm film can be shown by AFM. This result affects the significant properties. Figure 3c,d exhibit AFM profiles of 10 nm and 40 nm across the film edge, respectively.

### 3.2. Magnetic Property

**Figure 4:**
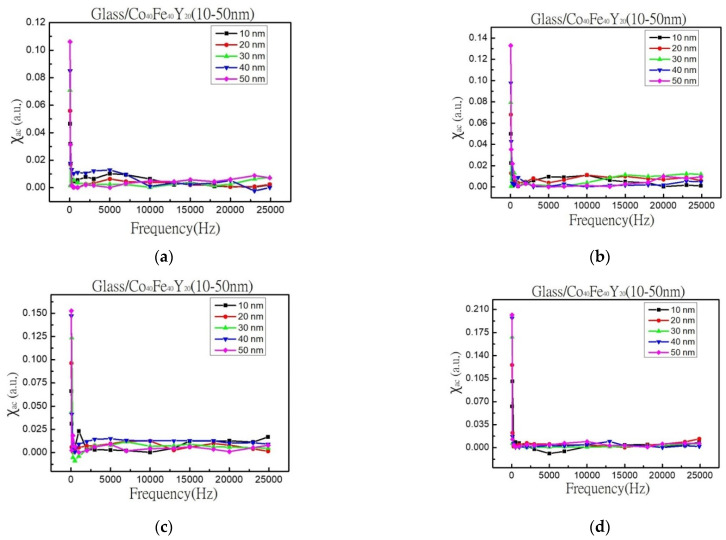
χ_ac_ result as a function of the frequency from 50 to 25,000 Hz. (**a**) RT, (**b**) after annealing at 100 °C, (**c**) after annealing at 200 °C, (**d**) after annealing at 300 °C.

The χ_ac_ is displayed in Figure 4a as a function of frequency from 50 Hz to 25,000 Hz for CoFeY films. The χ_ac_ of the annealed CoFeY samples are shown in Figure 4b–d versus a frequency ranging from 50 Hz to 25,000 Hz. The average error of alternate-current magnetic susceptibility measurement was ±0.005. The result demonstrates that χ_ac_ values declined as the measured frequency increased. The amplitude fell at high frequencies. Furthermore, because the glass substrate is diamagnetic, the negative signal in Figure 4c,d can be reasonably assumed to be the substrate signal or a small amount of noise. According to the result, the χ_ac_ value should be near zero at a high frequency. After annealing, the alternate-current magnetic susceptibility changed by magnetic annealing anisotropy [38].

**Figure 5:**
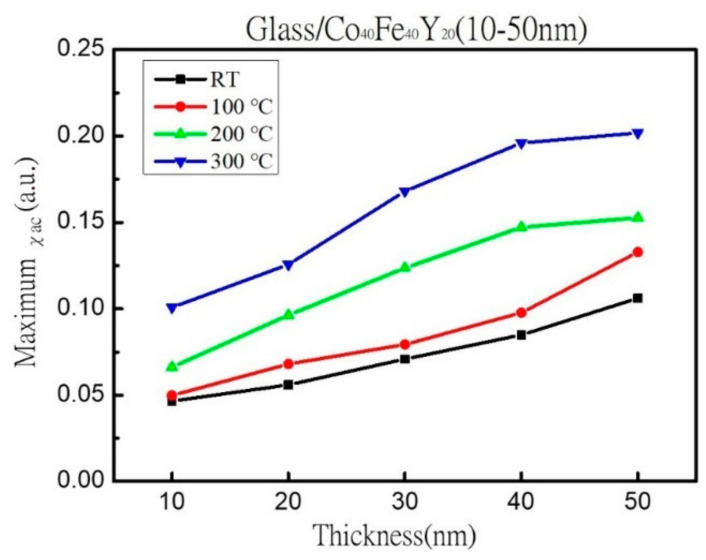
Maximum χ_ac_.

Figure 5 shows the corresponding maximum χ_ac_ values for as-deposited and three annealing temperatures with different thicknesses. This study shows that when thickness and annealing temperatures increased, the maximum χ_ac_ value increased as well. The results were obtained as a result of the thickness effect and Y addition, which increased the spin exchange coupling [39,40]. The thickness effect states that as the thickness increases, the maximum χ_ac_ values also increase [41]. When the thickness was 50 nm, the maximum χ_ac_ value was 0.11 at the as-deposited state, 0.13 when the annealed temperature was 100 °C, 0.15 when the heat treatment temperature was 200 °C, and the maximum χ_ac_ value was 0.20 when the heat treatment temperature was 300 °C. To investigate the effect of Y addition, the maximum χ_ac_ value of Co_40_Fe_40_Y_20_ film at 50 nm under the same four conditions is between 0.11 and 0.20, which is greater than the maximum χ_ac_ value of the previously studied Co_40_Fe_40_Y_10_B_10_ film of 0.05 to 0.16, implying that the Y addition can enhance spin-exchange coupling and induce higher values [42]. The data thus show that CoFeY films have a thickness effect [43,44]. The ideal resonance frequency (f_res_) denotes the frequency at which the maximum χ_ac_ has the greatest spin sensitivity. When the maximum χ_ac_ value was supplied in addition to the f_res_ value, the spin sensitivity improved considerably. The fact that the f_res_ values of the thin films were in the low-frequency range of 50 to 100 Hz provides more proof that CoFeY magnetic thin films can be used in soft magnetic devices.

**Figure 6:**
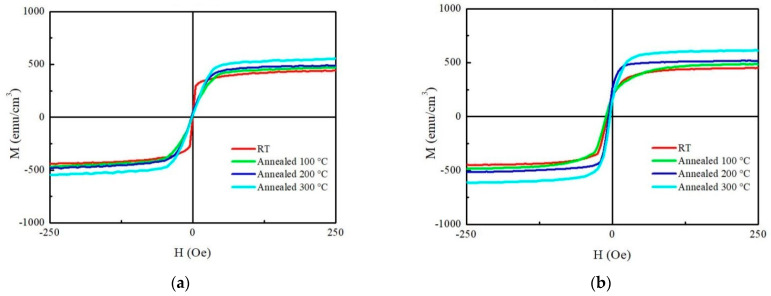
M-H loop of CoFeY films under four conditions; (**a**) 10 nm and (**b**) 50 nm.

Hysteresis loops are shown in Figure 6a,b for 10 nm and 50 nm to study magnetization (M) by an in-plane external field (H_ext_) field of 250 Oe. According to the M-H results, the Ms value at 10 nm is between 440 emu/cm^3^ and 545 emu/cm^3^, while the Ms value at 50 nm is between 482 emu/cm^3^ and 610 emu/cm^3^. The Ms of the annealed treatment is greater than the Ms of the as-deposited state. It also shows that the lower Hc and higher Ms indicate that CoFeY film is a promising material as a free or pinned layer for MTJ applications. Therefore, this magnetic characteristic is compatible with the χ_ac_ result. The modification of the shape of the hysteresis loop with an increase in the annealing temperature is seen due to magnetic anisotropy [45].

### 3.3. Electrical Properties

**Figure 7:**
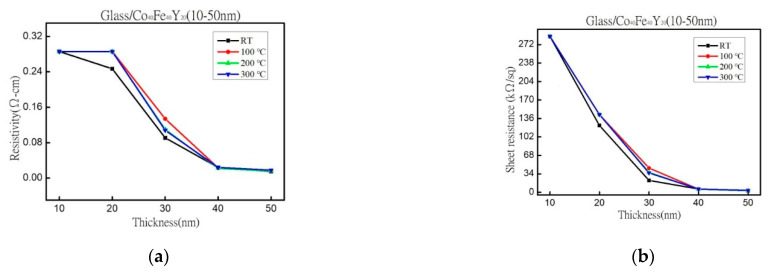
(**a**) The resistivity. (**b**) The sheet resistance.

The resistivity of various thicknesses and at four conditions is depicted in Figure 7a. Figure 7b displays the sheet resistance for four different states and various thicknesses. When the film was thicker, the resistivity was smaller, and the amplitude of the resistance was closer to being flat. Because electron carriers are less constrained in their conduction, the electric resistivity and sheet resistance of thick film thickness and higher annealing temperatures are smaller than those of thinner thicknesses and lower annealing temperatures. The reason for the thinner film may be that it is not a continuous film throughout growth, which causes more barriers to electron transport and an increase in resistivity. It indicates that increasing the film thickness and fewer obstacles hinder the movement of electrons and increase electron conductivity [46,47]. For the 30 nm film, the resistivity is minimum at RT, and the resistivity is maximum for the 100 °C annealed film because the film is very thin, and it may be caused by the growth mode of the discontinuous film.

### 3.4. Analysis of Surface Energy and Adhesion

**Figure 8:**
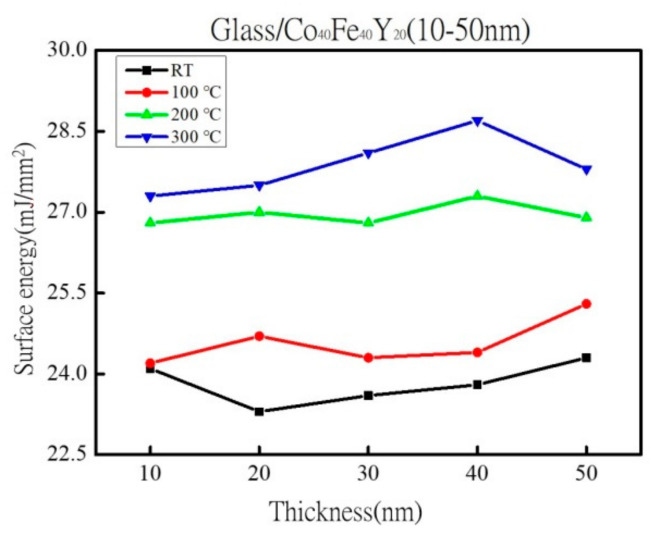
The surface energy under four conditions.

Figure 8 displays the surface energy for four distinct scenarios where the film thickness was raised from 10 nm to 50 nm. In comparison to the freshly as-deposited films, annealed films have a larger surface energy. The surface energy with the greatest value was 28.7 mJ/mm^2^ when the film was annealed at 300 °C with a thickness of 40 nm. It was found that the film adhered to surfaces with a greater force as the surface energy increased. Additionally, it was shown that the contact angle and surface energy have a close relationship. A greater liquid absorption area and a decrease in the contact angle occur when the surface free energy is high [48]. More liquid is also absorbed when the surface free energy is high. The contact angle and Young’s equation are used to derive the surface energy [29,30,31]. Low surface energy is correlated with weak adhesion [49]. The adhesion was strongest when the surface energy of the films was higher. These findings suggest that it is simpler to combine with the MTJ structure’s layer. The surface energy range of CoFeY films is from 23.3 mJ/mm^2^ to 28.7 mJ/mm^2^, which is more than fluorinated ethylene propylene copolymer (FEP) and equivalent to polystyrene (PS) [50].

### 3.5. Analysis of Optical Property

Table 4 displays the maximum and minimum transmittance in as-deposited and annealed states. It shows a decrease in minimum transmittance from 65.1% to 21.4% between the thicknesses of 10 and 50 nm. According to Table 4, the minimum transmittance dropped from 66.5% to 20.1% at an annealed temperature of 100 °C. At the 200 °C annealing temperature, it showed that the minimum transmittance dropped from 66.7% to 22.4%. Table 4 demonstrates that at the 300 °C annealing temperature, the minimum transmittance dropped from 70.7% to 20.8%. The annealing temperature was raised, but only a very slight change in transmittance was seen, and the trend was not immediately obvious. With increasing film thickness, the absorption strength of Co_40_Fe_40_Y_20_ films increased, but the transmittance decreased. This is due to a decrease in transmittance caused by the thickness effect, which mutes the optical signal [51]. It is assumed that when the thickness of most materials increases, the transmittance decreases. The film may hinder photons from passing through and reduce transmittance due to its thickness and high annealing temperature. The effect of annealing temperature on hydrophilicity and optical transmittance properties indicates that the contact angle results at the annealing temperature are smaller than that of the as-deposited film, which can be reasonably concluded that there is an increased continuous film mode after annealing. Moreover, the transmittance after annealing is slightly lower than RT, which means that continuous film occurs at a high annealing temperature, resulting in a slight decrease in the transmittance. Due to the state of the continuous film, the values of surface energy and magnetic characteristics are higher at a thick film thickness and higher annealing temperatures than they are at thinner thicknesses and lower annealing temperatures.

### 3.6. Hardness and Young’s Modulus

**Figure 9:**
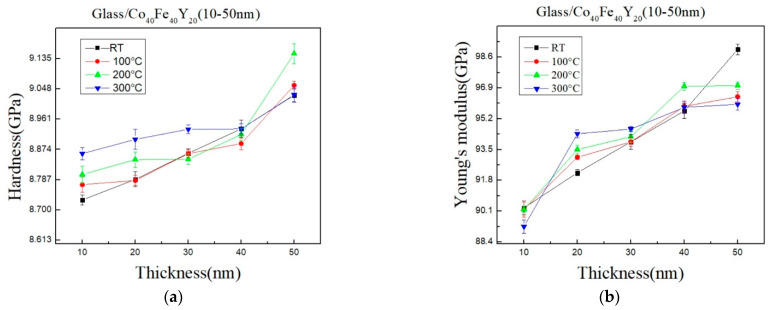
Nano-indentation of CoFeY thin films. (**a**) Hardness and (**b**) Young’s modulus.

Figure 9a,b shows the hardness (H) and Young’s modulus (E) in their as-deposited and annealed states, respectively. When the thickness is increased, the hardness and Young’s modulus increase as a result. A nano-hardness indentation can be determined using the Pharr–Oliver method, which is based on the loading and unloading curve and exposes the combined hardness of the glass substrate and the CoFeY thin film [52]. Additionally, similar outcomes were also obtained with tungsten (W) films grown on glass and silicon substrates with an indentation displacement twice as great as the film thickness [53]. The substrate effects are significant even at shallow indentation depths. Given the thinness of the CoFeY thin film, it is reasonable to assume that the substrate will have an impact on the nanoindentation measurement. Furthermore, it can be found that the Young’s modulus maximum at RT and the hardness maximum at 200 °C because the initial pop-in displacement might be connected to the material’s yielding point [54]. However, it can be challenging to pinpoint the initial displacement burst since it may occur early in the loading process as a result of instrument error or surface roughness. At 200 °C for 1 h and 50 nm, the hardness achieves the maximum value, and further annealing reduces the hardness. The same behavior in terms of hardness was seen [55]. Generally, annealed films are harder than as-deposited films because annealing reduces free volume concentration, shortens the interatomic distance, and increases hardness [56,57]. According to the surface energy and hardness results, the films have changed after annealing due to atomic arrangement by thermal driving force [49].

## 4. Conclusions

In XRD, an amorphous state was identified due to insufficient thermal driving force and Y addition. According to the alternating current susceptibility experiment, the maximum χ_ac_ value was obtained at 50 nm and annealed at 300 °C. The highest χ_ac_ value of the Co_40_Fe_40_Y_20_ film increased following heat treatment. Due to the thickness effect and Y addition, the maximum χ_ac_ value of each CoFeY film grew as film thickness increased, enhancing the spin exchange coupling. The f_res_ means that the maximum χ_ac_ has the highest spin sensitivity in the range of 50-100 Hz at all conditions. In comparison to thinner thicknesses and lower annealing temperatures, thick film thickness and higher annealing temperatures have lower electric resistivity and sheet resistance due to electron carrier movement. The surface energy increased as the heat treatment temperature increased. The maximum surface energy was obtained after annealing the 40 nm-thick film at 300 °C with a surface energy of 28.7 mJ/mm^2^. With increasing thickness, the hardness and Young’s modulus increase. The hardness reaches its maximum value after one hour at 200 °C and 50 nm, and subsequent annealing reduces the hardness. According to optical measurements, the transmittance decreases with increasing thickness. The resistivity and optical transmission also decrease with the increase in film thickness in this study.

## Figures and Tables

**Table 1:** Names of proper nouns in full and abbreviated form.

Abbreviation	Full Name
CoFeY	Cobalt Iron Yttrium
Y	Yttrium
XRD	X-ray diffraction
χ_ac_	Low-frequency alternate-current magnetic susceptibility
Ms	Saturation magnetization
Hc	Coercivity
CoFe	Cobalt Iron
MRAM	Magnetic random-access memory
RE	Rare earth
Y_2_O_3_	Yttrium oxide
Co_40_Fe_40_B_20_	Cobalt Iron Boron
MTJ	Magnetic tunneling junction
RT	Room temperature
CoFeV	Cobalt Iron Vanadium
CoFeW	Cobalt Iron Tungsten
CoFeYb	Cobalt Iron Ytterbium
DC	Direct current
T_A_	Annealed temperature
Ar	Argon
GIXRD	Grazing incidence X-ray diffraction
EDX	Energy dispersive X-ray spectroscopy
AFM	Atomic force microscopy
a.u.	Arbitrary unit
ƒ_res_	Optimal resonance frequency
VSM	Vibrating sample magnetometer
θ	Contact angle
DI	Deionized
CSM	Continuous stiffness measurement
M	Magnetization
H_ext_	External field
FEP	Fluorinated ethylene propylene copolymer
PS	Polystyrene
W	Tungsten
H	Hardness
E	Young’s modulus

**Table 2:** Significant properties for CoFeV, CoFeW, and CoFeYb materials.

Materials	Maximum χ_ac_ (a.u.)	Surface Energy (mJ/mm^2^)	Transmittance (%)
Glass substrate/Co_40_Fe_40_V_20_ [23,24] 10–100 nm at RT	0.02–0.04	27.8–45.4	x
Glass substrate/Co_32_Fe_30_W_38_ [25] 10-50 nm at RT and annealed conditions	0.02–0.52	22.3–28.4	x
Glass substrate/Co_40_Fe_40_Yb_20_ [26] 10–50 nm at RT and annealed conditions	0.04–0.35	28.6–34.5	22.3–80.5
Si(100) substrate/Co_40_Fe_40_Y_20_ [27] 10–50 nm at RT and annealed conditions	0.03–0.16	22.7–31.1	x
Glass substrate/Co_40_Fe_40_Y_20_ 10–50 nm at RT and annealed conditions (Current research)	0.04–0.20	23.3–28.7	20.1–81.7

**Table 3:** EDX analysis data for alloy films.

Element	Weight%	Atomic%
Fe	28.06	36.56
Co	38.12	47.07
Y	33.82	16.37
Totals	100.00	

**Table 4:** Transmittance for Co_40_Fe_40_Y_20_ thin films.

Process	Thickness	Minimum Transmittance (%)	Maximum Transmittance (%)
As-deposited	10 nm	65.1	81.7
	20 nm	62.3	78.8
	30 nm	34.3	41.4
	40 nm	23.2	34.5
	50 nm	21.4	32.5
Annealing 100 °C	10 nm	66.5	78.6
	20 nm	58.7	74.6
	30 nm	39.1	42.3
	40 nm	24.2	31.2
	50 nm	20.1	29.4
Annealing 200 °C	10 nm	66.7	80.5
	20 nm	61.1	72.2
	30 nm	38.2	41.2
	40 nm	25.1	30.8
	50 nm	22.4	29.5
Annealing 300 °C	10 nm	70.7	78.4
	20 nm	60.1	66.6
	30 nm	31.1	36.1
	40 nm	22.4	29.1
	50 nm	20.8	28.2

## Data Availability

Not applicable.
